# Hydrolysis of woody biomass by a biomass-derived reusable heterogeneous catalyst†
†Electronic supplementary information (ESI) available: Experimental details, raw data of characterisation. See DOI: 10.1039/c5sc03377b


**DOI:** 10.1039/c5sc03377b

**Published:** 2015-10-15

**Authors:** Hirokazu Kobayashi, Hiroyuki Kaiki, Abhijit Shrotri, Kota Techikawara, Atsushi Fukuoka

**Affiliations:** a Institute for Catalysis , Hokkaido University , Kita 21 Nishi 10, Kita-ku , Sapporo , Hokkaido 001-0021 , Japan . Email: fukuoka@cat.hokudai.ac.jp; b Graduate School of Chemical Sciences and Engineering , Hokkaido University , Kita 13 Nishi 8, Kita-ku , Sapporo , Hokkaido 060-8628 , Japan

## Abstract

Biomass is the sole carbon-based renewable resource for sustaining the chemical and fuel demands of our future. Lignocellulose, the primary constituent of terrestrial plants, is the most abundant non-food biomass, and its utilisation is a grand challenge in biorefineries. Here we report the first reusable and cost-effective heterogeneous catalyst for the depolymerisation of lignocellulose. Air oxidation of woody biomass (*Eucalyptus*) provides a carbonaceous material bearing an aromatic skeleton with carboxylic groups (2.1 mmol g^–1^) and aliphatic moieties. This catalyst hydrolyses woody biomass (*Eucalyptus*) to sugars in high yields within 1 h in trace HCl aq. Furthermore, after the reaction, the solid residue composed of the catalyst and insoluble ingredients of woody biomass is easily transformed back to fresh catalyst by the same air oxidation method. This is a self-contained system using woody biomass as both the catalyst source and substrate for realising facile catalyst preparation and recycling.

## Abstract 2

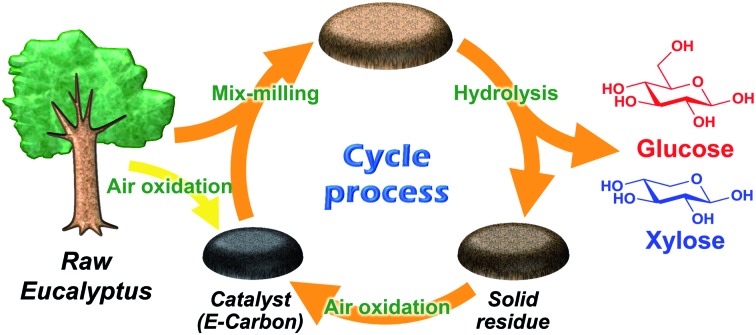
A carbon catalyst prepared by air oxidation of woody biomass hydrolyses woody biomass, and the reaction residue is transformed back to the catalyst by the same air oxidation method.

## Introduction

**Scheme 1:**
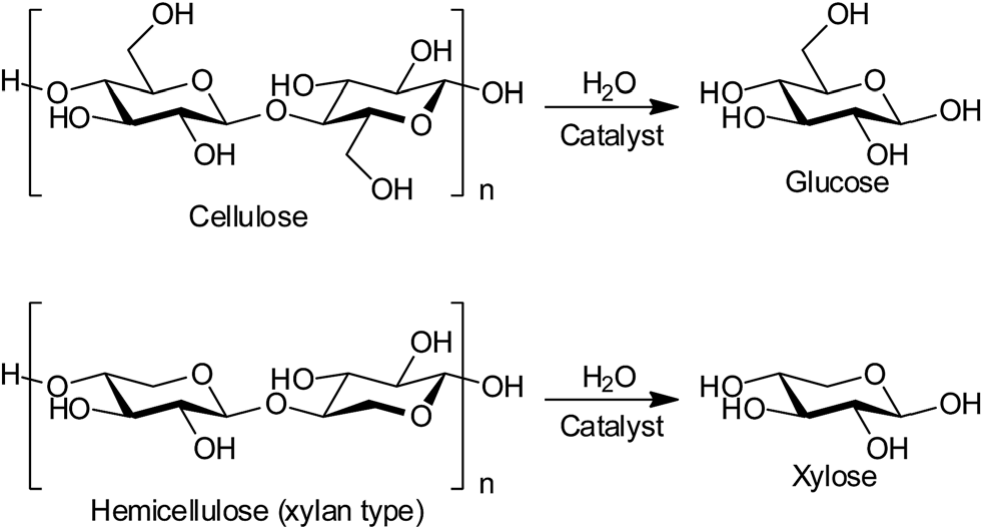
Hydrolysis of cellulose and hemicellulose fractions in lignocellulose.

Production of biofuels and bio-chemicals from lignocellulose, the most abundant non-food biomass, is a grand challenge in biorefineries.^1^–^3^ Lignocellulose is a composite of cellulose, hemicellulose and lignin, which are insoluble polymers of glucose, related sugars such as xylose, and phenyl propane derivatives, respectively. The hydrolysis of cellulose and hemicellulose to monomeric sugars (Scheme 1) is the first step in a biorefinery, and this reaction has been studied with both enzymes and chemical catalysts such as soluble mineral acids and solid acids.^1^,^4^ However, the practical use of these catalysts is hampered by reuse difficulties, high costs of preparation and disposal, and the need for long reaction times.^1^ Herein, we report the first reusable and cost-effective heterogeneous catalyst to resolve all these issues.

The use of heterogeneous catalysts is desired for the efficient depolymerisation of lignocellulose as they are non-corrosive and can be separated from product solution.^5^–^7^ It has recently been demonstrated that weakly acidic carbons^8^–^15^ and sulphonated carbons^16^–^23^ are active for the hydrolysis of cellulose as a model substrate. Carbon materials adsorb cellulosic molecules through CH–π hydrogen bonds and hydrophobic interactions,^24^,^25^ and then the adsorbed molecules are hydrolysed by acidic sites.^9^–^13^,^26^ The presence of defect sites on carbon may further improve the activity,^10^–^12^ as the adsorption of cellulosic molecules in confinement^13^ changes their conformation to weaken glycosidic bonds.^27^–^29^

Regardless of the preferable characteristics of heterogeneous catalysts, the contamination of the catalyst with solid lignin after the reaction prevents their application in the depolymerisation of real lignocellulose. Removal of lignin from solid catalysts is often challenging, essentially rendering the catalyst useless after the first reaction. Hence, the lignin fraction must be removed by pretreatment such as the kraft process before applying lignocellulose to the hydrolysis reaction.^9^ Another demerit of existing catalysts is the use of large amounts of chemicals such as bases and acids in creating active sites on the catalysts,^8^–^23^ leading to high costs and huge quantities of neutralisation waste in post-treatments (more than 50 kg of waste per 1 kg of catalyst; see ESI†).

Our idea for resolving the issues of conventional heterogeneous catalysts is to produce a weakly acidic carbon catalyst through simple air oxidation of lignocellulose and lignin residue. Organic materials thermally decompose to form carbonaceous material at an elevated temperature^30^ and simultaneously gain weakly acidic oxygenated groups (active sites) in the presence of air *via* oxidation. Thus, we can expect that the carbon material prepared by air oxidation hydrolyses lignocellulose. In this way, the catalyst is readily prepared, and more importantly the used catalyst and residual lignin can be together transformed into fresh catalyst by the same air oxidation method.

## Results and discussion

### Whole scheme of our system

**Fig. 1:**
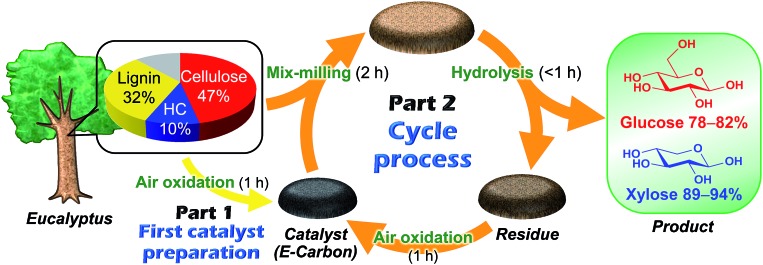
Schematic representation of the *E-Carbon* system. HC: xylan hemicellulose.

Fig. 1 represents our system using air oxidation for hydrolysis of lignocellulose. The first part (Part 1) is the production of a carbon-based catalyst from *Eucalyptus* by air oxidation, which is necessary only once. The second part (Part 2) is a cyclic process consisting of milling pretreatment, hydrolysis of *Eucalyptus* to glucose and xylose in trace HCl, and transformation of the solid residue to fresh catalyst by the same air oxidation. We used *Eucalyptus* as both a catalyst source and biomass substrate to make this a self-contained system. *Eucalyptus* is a fast-growing and inexpensive plant [<0.1 pounds (GBP) kg^–1^] that has been cultured as a major feedstock for pulping.^31^,^32^

### Preparation and characterisation of the catalyst

We first studied the initial air oxidation of *Eucalyptus* to produce a catalyst. *Eucalyptus* powder was first washed with boiling water and dried (see ESI†). The resulting solid was used in all the experiments. The major ingredients of the washed *Eucalyptus* powder were cellulose (47 wt%), xylan hemicellulose (10 wt%) and lignin (32 wt%). Ash was present in a small amount (0.12 wt%) and mainly composed of Ca salts (Table S1†). The air oxidation of the *Eucalyptus* powder was performed at 573 K for 1 h, which were the optimised conditions for preparing an active catalyst (Table S2†). This procedure gave a black material (named *E-Carbon*, Fig. S1†) in 49% yield based on the carbon content. The remaining part was lost as CO, CO_2_ and volatile organic compounds such as tar. The maximum temperature inside the sample was 575 K during the process, showing no formation of hot spots.

**Fig. 2:**
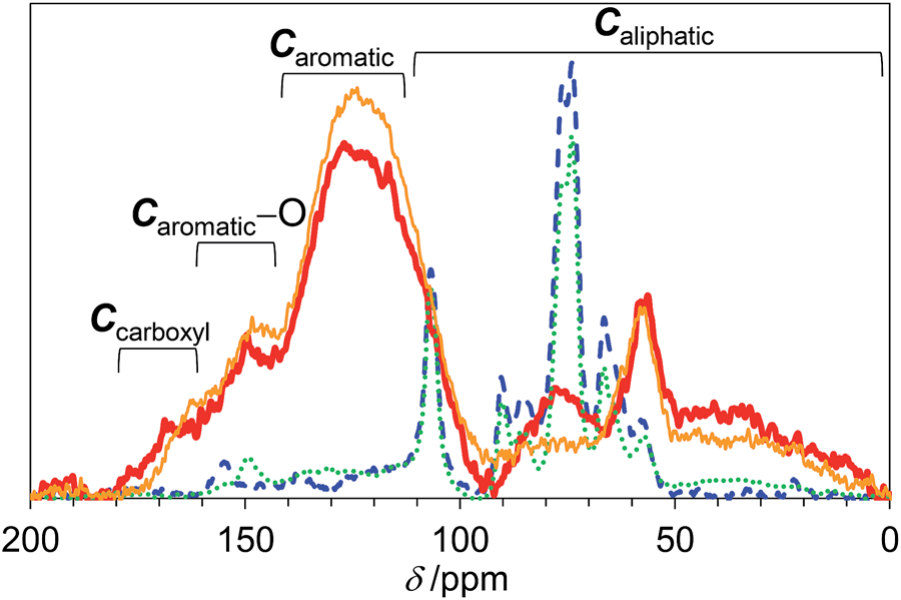
^13^C CP/MAS NMR spectra of *E-Carbon* (red bold solid line), recycled *E-Carbon* (orange narrow solid line), N_2_-treated *Eucalyptus* (green dotted line) and pristine *Eucalyptus* (blue dashed line).

**Fig. 3:**
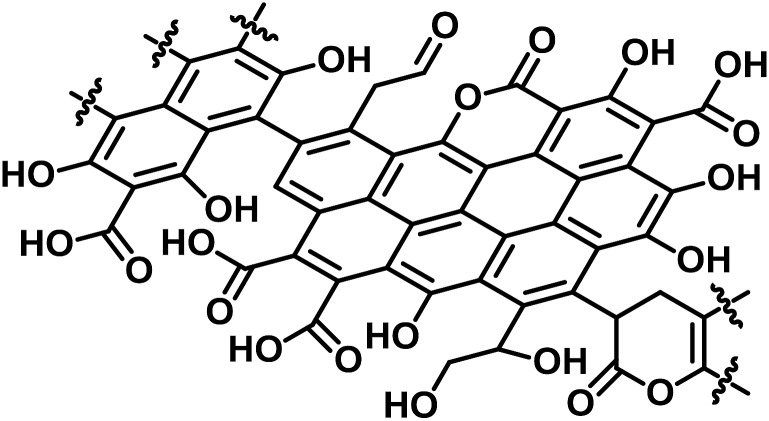
Proposed structure of *E-Carbon*.

The chemical structure of *E-Carbon* was determined with several physicochemical techniques. Solid-state ^1^H–^13^C cross polarisation/magic angle spinning nuclear magnetic resonance (CP/MAS NMR) was used to clarify the structural change of *Eucalyptus* by air oxidation (Fig. 2). The pristine *Eucalyptus* (blue dashed line) gave major peaks at 110–50 ppm, mainly ascribed to cellulose and hemicellulose.^33^ Small NMR signals at ≥110 ppm are derived from lignin (*C*_aromatic_–O at 160–140 ppm, *C*_aromatic_–C and *C*_aromatic_–H at 140–110 ppm).^34^–^36^ After air oxidation (red solid line), a predominant peak appeared at 125 ppm due to the formation of non-oxygenated aromatic carbons. Smaller broad peaks were observed in the regions for –*C*O_2_R (170 ppm), *C*_aromatic_–O (150 ppm) and sp^3^ carbons (<100 ppm).^34^,^35^ Infrared (IR) measurement of *E-Carbon* in a transmission mode (Fig. S2†) showed four major peaks ascribed to *ν*(C=O) (1770–1720 cm^–1^), *ν*(C=C, aromatic) (1610 cm^–1^), *δ*(C–H) (1470–1370 cm^–1^) and a mixture of various vibrations such as *ν*(C–O) (1350–1000 cm^–1^),^37^ indicating the presence of aromatic rings and oxygenated groups. X-ray photoelectron spectroscopy (XPS) in the C 1s region represented a quantitative distribution of functional groups: –CO_2_R at 288.6 eV (13 ± 1%), C=O at 287.2 eV (3 ± 2%), C–O at 286.2 eV (20 ± 5%), and C–C and C=C at 284.6 eV (65 ± 5%) (Fig. S3†).^38^ The content of carboxylic acid in *E-Carbon* was 2.1 mmol g^–1^, determined by a titration experiment with NaHCO_3_.^39^ Elemental analysis of *E-Carbon* showed that the amounts of C, H, N and O were 62.5 wt%, 2.2 wt%, <0.3 wt% and 35.0 wt%, respectively, where the oxygen content was estimated by subtracting the weight of C, H and ash from 100 wt%. The ratio corresponds to CH_0.43_O_0.42_. *E-Carbon* adsorbed a large amount of water (1.8 mmol g^–1^ at *p*/*p*_0_ = 0.1; Fig. S4†) at 298 K, which was *ca.* 160 times greater than the adsorption amount of N_2_ at 77 K at the same relative pressure (Fig. S5†). This result indicates the condensation of water in *E-Carbon* due to a high concentration of oxygenated groups.^40^ The Raman spectrum of *E-Carbon* contained a broad D-band at 1390 cm^–1^ and a G-band at 1590 cm^–1^ (*I*_G_ > *I*_D_; Fig. S6†), which are characteristic of amorphous carbon materials with polycyclic aromatics.^41^,^42^ The amorphous structure was also indicated by X-ray diffraction measurements (Fig. S7†). Hence, we concluded that *E-Carbon* consists of an aromatic framework with weakly acidic groups and aliphatic moieties (Fig. 3, the composition is CH_0.44_O_0.42_). It was confirmed that the aromatics and acidic sites were not only derived from lignin but also from cellulose fractions; air oxidation of cellulose gave a similar carbon material (named air-oxidised cellulose, see Fig. S2†).

We also prepared a catalyst by heat-treatment of *Eucalyptus* under N_2_ at 573 K as a control. This material had significantly weaker aromatic peaks in the NMR (green dotted line in Fig. 2) and IR spectra (Fig. S2†). Accordingly, our results show that air oxidation provides more aromatics than N_2_ treatment at 573 K. This is reasonable as the air oxidation of organic polymers (*e.g.*, polyacrylonitrile) gives aromatic precursors at 473–573 K for the manufacture of carbon fibres.^43^ As for the oxygenated groups in N_2_-treated *Eucalyptus*, only weak C=O peaks were observed in the IR spectrum (Fig. S2†) and the area percentage of –CO_2_R was only 2 ± 1% in the XPS (Fig. S3†). The specific amount of carboxylic acid groups determined by titration was 0.11 mmol g^–1^, corresponding to 1/20 of that of *E-Carbon*. Clearly, air oxidation is essential for introducing a large amount of carboxylic acid groups onto the carbons.

### Pretreatment and catalytic reaction

*E-Carbon* and *Eucalyptus* [1 : 6.48 (wt)] were milled together, named mix-milling,^9^ by planetary ball-milling^44^–^46^ for realising a high-yielding synthesis of sugars. Cellulose and hemicellulose are encapsulated by lignin in lignocellulose for protecting the carbohydrates from chemical attack, but milling treatment ruptures the composite to expose the sugar polymers.^47^ More importantly, specific to the mix-milling, good contact between the solid catalyst and solid substrate are created at the same time.^48^ This treatment required only 2 h for improving the reaction performance in the present study. Although this optimisation using planetary ball-milling is for laboratory-scale experiments, large-scale milling is applicable to maximise the economic efficiency in industry.^45^,^47^

The mix-milled solid containing *E-Carbon* (50 mg) and *Eucalyptus* (324 mg) was subjected to a hydrolysis reaction in a 120 ppm HCl aqueous solution (pH 2.5) at 488 K (Table 1). This reaction gave glucose in 78% yield based on the carbon content of cellulose in *Eucalyptus* (Table 1, entry 4). The hemicellulose fraction of *Eucalyptus* was also hydrolysed to xylose in 94% yield. Thus, both cellulose and hemicellulose can be utilised in this system. The major by-products were oligosaccharides, sugar isomers, 5-hydroxymethylfurfural, levoglucosan and furfural (detailed results are shown in Table S3†). The total amount of the sugars and soluble by-products was almost consistent with the carbohydrate content in the *Eucalyptus* sample (carbon balance: 96%).

**Table 1:** Hydrolysis of *Eucalyptus* by *Eucalyptus*-based catalysts^*a*^

Entry	Solvent	Catalyst	Product yield/%	
			Glucose^*b*^	Xylose^*c*^
1	Water	No catalyst	3	30
2	Water	*E-Carbon*	31	83
3	HCl^*d*^	No catalyst	32	26
4	HCl^*d*^	*E-Carbon* (1st cycle)	78	94
5	HCl^*d*^	*E-Carbon* (2nd cycle)	82	89
6	HCl^*d*^	N_2_-treated *Eucalyptus*	28	26
7	HCl^*d*^	Air-oxidised cellulose	77	91

^*a*^Reaction conditions: mix-milled sample 374 mg [*Eucalyptus* 324 mg, catalyst 50 mg (containing 0.11 mmol of carboxylic acid groups)], solvent 40 mL. The reaction temperature was elevated from 298 K to 488 K in 17 min, and then quickly cooled down to 298 K.

^*b*^Based on carbon content of cellulose.

^*c*^Based on carbon content of xylan.

^*d*^120 ppm HCl (= 0.13 mmol).

In the hydrolysis of *Eucalyptus*, we assume that *E-Carbon* and the mild acidic solvent (pH 2.5) synergistically accelerate the formation of monomeric sugars. It has been reported that trace HCl hydrolyses cellulose to produce soluble oligosaccharides, which enables the subsequent hydrolysis of oligosaccharides by solid acid catalysts.^49^ In contrast, the roles of solid catalyst and HCl are reversed in our system due to the mix-milling.^9^ Weakly acidic carbons quickly hydrolyse solid cellulose to soluble oligosaccharides owing to the close contact created by mix-milling. As a result, the hydrolysis of oligosaccharides is the rate-determining step, which needs to be accelerated by soluble acid to maximise yields of monomeric sugars. Indeed, the hydrolysis of cellulose by a carbon catalyst in water after mix-milling almost completely converted cellulose (93%) with soluble oligomers as the main product (70% yield), whereas hydrolysis by HCl gave a low conversion of cellulose (39%) with the formation of glucose as a main product (27% yield).^9^ Therefore, in the conversion of *Eucalyptus*, reactions in the absence of HCl or carbon catalyst provide unpractical yields of monomeric sugars (entries 1–3). HCl can be neutralised after the reaction with very low economic impact, as the acid concentration is less than 1/50 of conventional mineral acid processes.^1^

Controlled experiments were performed to reveal the important parameters influencing the catalytic activity of *E-Carbon*. Since a reaction in aq. HCl without *E-Carbon* afforded glucose in 32% yield and xylose in 26% yield (entry 3), *E-Carbon* increases the yield of glucose by 46% and yield of xylose by 68% (subtraction of yields in entry 3 from those in entry 4). The increase corresponds to a turnover number of carboxylic acid of 5.6. The result indicates that *E-Carbon* acts as a catalyst for the hydrolysis of cellulose and hemicellulose in *Eucalyptus*. We also found that the air-oxidised carbon prepared from cellulose worked in this reaction similarly to *E-Carbon* as shown in entry 7 (glucose 77%, xylose 91%). This shows that a cellulose-derived part also constitutes the active catalytic domain in *E-Carbon*. Contrastingly, the *Eucalyptus*-based catalyst prepared by N_2_ treatment was inactive (glucose 28%, xylose 26%; entry 6). Therefore, it is concluded that the air oxidation of woody biomass provides active catalysts for the hydrolysis of lignocellulose.

The solid residue recovered after the reaction with *E-Carbon* can be converted to a fresh catalyst again by air oxidation as shown in Fig. 1. This is the outstanding characteristic of our catalyst, since all the previous carbon-based catalysts were single use in the hydrolysis of raw biomass.^8^–^23^ The treatment at 573 K for 1 h converted the solid residue of 1.63 g [1.12 g derived from *Eucalyptus* (mainly lignin) and 0.51 g of *E-Carbon*, obtained in a large-scale experiment] to a black powder of 1.14 g. Accordingly, the catalyst weight increased from 0.51 to 1.14 g after one cycle in this system. The surplus residue can be used as fuel to power the process, since the solid is derived only from woody biomass and air. The ^13^C CP/MAS NMR spectrum of the regenerated catalyst contained a strong aromatic carbon peak with small fractions of –*C*O_2_R, *C*_aromatic_–O and aliphatic groups (Fig. 2, orange solid line). This character is similar to that of original *E-Carbon*. A portion of the prepared catalyst was again mix-milled with *Eucalyptus* and subjected to the hydrolysis reaction in the same manner as described above. The reaction produced glucose in 82% yield and xylose in 89% yield (Table 1, entry 5). We also compared the catalytic activity of the first- and second-cycle *E-Carbon* at a lower temperature. The experiments at 473 K indicated no decline of catalytic activity by the second air oxidation (Table S4†). It is thus demonstrated that the mixture of lignin and *E-Carbon* changes to a new active *E-Carbon* by air oxidation. Since both lignin and *E-Carbon* are aromatic polymers, their transformation to the new catalyst is easier than the first synthesis of *E-Carbon* from lignocellulose. Our system can leverage the contaminant (lignin) for the preparation of catalyst, which is in sharp contrast to conventional catalytic processes that require removal of the contaminant.

Comparing *E-Carbon* with reported catalysts, the active site is slightly similar to those of enzymes (cellulase).^50^ Both of them do not use strong acid but involve weak acids (carboxylic acids). However, they have different optimal conditions due to the difference in their skeletal and active structures. The carbon is composed of an aromatic framework and weak acids, and thereby high temperatures and a wide range of pH are applicable in catalytic reactions. On the other hand, cellulase is a protein that requires a conjugated base (carboxylate) in addition to carboxylic acid for the dissociation of glycosidic bonds; thus, low temperature and careful control of the pH with a buffer are necessary to keep the enzyme active. Consequently, *E-Carbon* can work under harsher conditions, which enables the rapid hydrolysis of lignocellulose in trace HCl aq. at high temperature. Moreover, our catalyst is reusable and the price (*ca.* 0.1 GBP kg^–1^) is two-orders lower than that of cellulase (6.5–26 GBP kg^–1^).^1^,^51^

## Conclusions

The air oxidation of biomass feedstock, *Eucalyptus*, produces a carbon-based catalyst overcoming the limitations of conventional catalysts used for the hydrolysis of woody biomass. The catalyst quickly converts lignin-containing *Eucalyptus* to glucose and xylose in high yields. Lignin remains as a solid together with the catalyst after the reaction; however, this solid mixture is a source for fresh catalyst and fuel. Therefore, the *E-Carbon* system drastically reduces the preparation and post-treatment costs of the catalyst. In general, the deactivation or spoiling of catalyst by contaminant is often a major issue in catalytic reactions. Hence, our idea that converts contaminant to a catalyst can be a useful strategy for improving the efficiency of catalytic processes.

## Methods

### Preparation of *E-Carbon*

*Eucalyptus* powder was washed with boiling water prior to use for all purposes in this study. 4.00 g of dried *Eucalyptus* powder was spread with a thickness of 3 mm on a Pyrex dish (*ø*130) to uniformly prepare the catalyst and avoid hot spots. The sample was calcined under air at atmospheric pressure in an electric furnace with the following program: 298 to 573 K by 5 K min^–1^ and 573 K for 1 h. In the case of reaction residue, the residue of 1.63 g was calcined under the same conditions. The temperature inside the sample was monitored using a thermocouple (*ø*0.5) equipped with a quartz tube (*ca. ø*1).

### Mix-milling

*Eucalyptus* (5.0 g) and catalyst (0.77 g) were milled together in an Al_2_O_3_ pot (250 mL) with Al_2_O_3_ balls (99.9%, *ø*15, 210 g) using a Fritsch P-6 planetary ball mill. Milling conditions were 500 rpm for 2 h with a 10 min interval after every 10 min of milling.

### Catalytic reaction

The hydrolysis of *Eucalyptus* was performed in a hastelloy C-22 high-pressure reactor equipped with an agitator operating at 600 rpm and a thermocouple. Mix-milled sample (374 mg) and 40 mL of 120 ppm HCl aq. were added into the reactor. The temperature of the reaction mixture was elevated from 298 K to 488 K in *ca.* 17 min and then quickly lowered to 298 K. Soluble products were analysed by high-performance liquid chromatography (Shodex SUGAR SH1011 and Phenomenex Rezex RPM-Monosaccharide Pb++ columns with refractive index detectors).
